# The Acceptability and Feasibility of Implementing a Bio-Behavioral Enhanced Surveillance Tool for Sexually Transmitted Infections in England: Mixed-Methods Study

**DOI:** 10.2196/publichealth.9010

**Published:** 2018-05-04

**Authors:** Sonali Wayal, David Reid, Paula B Blomquist, Peter Weatherburn, Catherine H Mercer, Gwenda Hughes

**Affiliations:** ^1^ Centre for Population Research in Sexual Health and HIV Institute for Global Health University College London London United Kingdom; ^2^ Centre for Infectious Disease Surveillance and Control HIV & STI Department Public Health England London United Kingdom; ^3^ The National Institute for Health Research Health Protection Research Unit in Blood Borne and Sexually Transmitted Infections University College London / Public Health England / London School of Hygiene & Tropical Medicine London United Kingdom; ^4^ Sigma Research Public Health, Environments and Society London School of Hygiene & Tropical Medicine London United Kingdom

**Keywords:** public health surveillance, sexually transmitted diseases, feasibility studies, electronic health records, Web-based survey

## Abstract

**Background:**

Sexually transmitted infection (STI) surveillance is vital for tracking the scale and pattern of epidemics; however, it often lacks data on the underlying drivers of STIs.

**Objective:**

This study aimed to assess the acceptability and feasibility of implementing a bio-behavioral enhanced surveillance tool, comprising a self-administered Web-based survey among sexual health clinic attendees, as well as linking this to their electronic health records (EHR) held in England’s national STI surveillance system.

**Methods:**

Staff from 19 purposively selected sexual health clinics across England and men who have sex with men and black Caribbeans, because of high STI burden among these groups, were interviewed to assess the acceptability of the proposed bio-behavioral enhanced surveillance tool. Subsequently, sexual health clinic staff invited all attendees to complete a Web-based survey on drivers of STI risk using a study tablet or participants’ own digital device. They recorded the number of attendees invited and participants’ clinic numbers, which were used to link survey data to the EHR. Participants’ online consent was obtained, separately for survey participation and linkage. In postimplementation phase, sexual health clinic staff were reinterviewed to assess the feasibility of implementing the bio-behavioral enhanced surveillance tool. Acceptability and feasibility of implementing the bio-behavioral enhanced surveillance tool were assessed by analyzing these qualitative and quantitative data.

**Results:**

Prior to implementation of the bio-behavioral enhanced surveillance tool, sexual health clinic staff and attendees emphasized the importance of free internet/Wi-Fi access, confidentiality, and anonymity for increasing the acceptability of the bio-behavioral enhanced surveillance tool among attendees. Implementation of the bio-behavioral enhanced surveillance tool across sexual health clinics varied considerably and was influenced by sexual health clinics’ culture of prioritization of research and innovation and availability of resources for implementing the surveys. Of the 7367 attendees invited, 85.28% (6283) agreed to participate. Of these, 72.97% (4585/6283) consented to participate in the survey, and 70.62% (4437/6283) were eligible and completed it. Of these, 91.19% (4046/4437) consented to EHR linkage, which did not differ by age or gender but was higher among gay/bisexual men than heterosexual men (95.50%, 722/756 vs 88.31%, 1073/1215; *P*<.003) and lower among black Caribbeans than white participants (87.25%, 568/651 vs 93.89%, 2181/2323; *P*<.002). Linkage was achieved for 88.88% (3596/4046) of consenting participants.

**Conclusions:**

Implementing a bio-behavioral enhanced surveillance tool in sexual health clinics was feasible and acceptable to staff and groups at STI risk; however, ensuring participants’ confidentiality and anonymity and availability of resources is vital. Bio-behavioral enhanced surveillance tools could enable timely collection of detailed behavioral data for effective commissioning of sexual health services.

## Introduction

### Sexually Transmitted Infections’ Surveillance in England

Globally, the burden of sexually transmitted infections (STI) continues to be high [1,2]. STI surveillance is a valuable public health tool to monitor the scale and trends of infections and the effectiveness of prevention strategies. In England, Public Health England (PHE), an executive agency of the Department of Health, manages a mandatory, national STI surveillance system known as GUMCAD, which contains pseudonymized, patient-level, electronic health records (EHR) of STI diagnoses and sexual health services accessed by all sexual health clinic (SHC) attendees, along with their sociodemographic characteristics [3]. PHE is also responsible for providing guidance on the management of STI outbreaks and epidemics [4]. Recent GUMCAD data have shown that between 2007 and 2016, the number of new STI diagnoses in England has increased [5]. Men who have sex with men (MSM), young people, and people of black ethnic minorities, particularly black Caribbeans (BC), bear a disproportionate STI burden and thus are priority groups for STI prevention efforts. Although existing GUMCAD data provide an excellent overview of STI epidemics and variations in subgroups, its interpretation is hampered by the lack of systematically collected information on STI risk behaviors. Enhancements to GUMCAD by collecting data on behavioral indicators of STI risk are planned [6], but these will not (and are not intended to) provide sufficient detail to investigate risk practices and contextual factors associated with neither specific nor evolving epidemics.

### Collecting Biological and Behavioral Data for Surveillance of Sexually Transmitted Infections

In the context of HIV, globally, since the late 1990s, tailoring surveillance to the epidemic state of a country and collecting and comparing behavioral and prevalence data have helped better understand the course of epidemics [7]. Similarly, in England, an investigation of sexually transmitted *Shigella fl* *exneri* outbreak in MSM between 2012 and 2013 using face-to-face semistructured quantitative interviews uncovered unexpected risk behaviors and their drivers, and highlighted the value of collecting enhanced behavioral data alongside biological data for informing STI control strategies in response to emergent public health concerns [8]. Thus, a bio-behavioral enhanced surveillance tool (BBEST) could be designed to collect detailed behavioral, attitudinal, and contextual data, depending on the nature of the public health concern, that is, data that will not be available in GUMCAD. However, the acceptability and feasibility of using a BBEST to collect sensitive behavioral data, especially if these relate to illicit behaviors, for example, recreational drug use, from SHC attendees and linking these to EHRs for STI surveillance are unknown. Moreover, peoples’ ability and willingness to accurately respond to sensitive behavioral questions could influence the reliability and validity of such data. Compared with face-to-face and with pen-and-paper interviews, computer-assisted self-interview (CASI) methods have been shown to result in greater disclosure of sensitive behaviors [9]. CASI also reduces item nonresponse, in part due to programmed routing of questions [10], thereby increasing the validity of resulting parameters [11].

### Study Aims and Objectives

Our aim was to assess the acceptability and feasibility of implementing a BBEST, comprising offering a self-administered Web-based survey to SHC attendees in England, to be completed using digital devices, and subsequently linking their survey responses to their EHR. Although we undertook this assessment in SHCs, our primary focus was on people of BC ethnicity and MSM as exemplar populations because of high STI burden among these groups, as mentioned previously.

## Methods

### Study Overview

**Figure 1:**
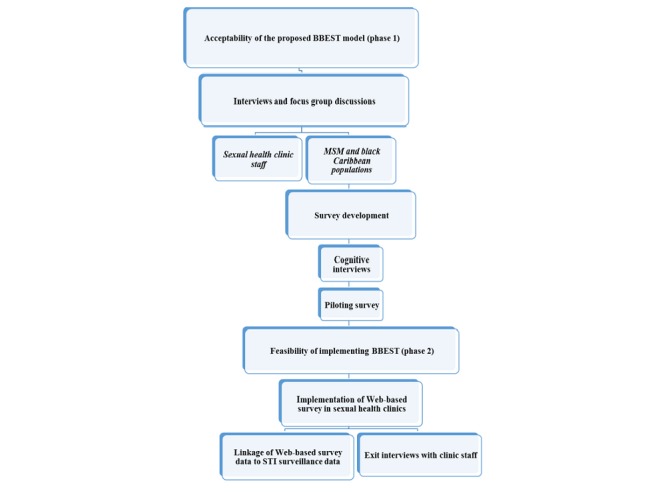
Study design for developing and implementing the bio-behavioral surveillance tool (BBEST) for sexually transmitted infections. MSM: men who have sex with men; STI: sexually transmitted infection.

We used a mixed methods study design (Figure 1) comprising phase 1 to assess acceptability of the proposed model of BBEST and phase 2 to examine the feasibility of implementing it. A Community Advisory Group (CAG) and a Steering Group comprising experts and stakeholders in the field of sexual health were set up to guide the study process. The CAG was involved in the development of study materials, including developing study posters, participant information sheet (PIS), and the terminology used in the survey. National Research Ethics Service Committee of South Central-Oxford C approved the study (reference: 15/SC/0223). This research was undertaken as part of the National Institute of Health Research Health Protection Research Unit (NIHR HPRU) in Blood-Borne and Sexually Transmitted Infections at University College London.

### Setting

Due to dearth of data on contextual drivers of STI among MSM and BC populations [12,13], informed by 2014 GUMCAD data, we decided to implement the BBEST in SHCs that had a high proportion of BC attendees (n=13) and MSM attendees (n=3), referred to henceforth as “BC only study sites” and “MSM only study sites,” respectively. Additional 3 SHCs with high proportions of both BC and MSM attendees were selected and are referred to henceforth as “combined study sites.”

### Phase 1: Assessing the Acceptability of the Bio-Behavioral Enhanced Surveillance Tool

#### Proposed Model of Bio-Behavioral Enhanced Surveillance Tool

We anticipated that the implementation of the BBEST in SHCs would involve the staff offering study envelopes and digital tablets to clinic attendees to participate in a Web-based survey. Participants could also use their personal digital device for survey completion. The survey would be administered using the Snap software (Snap Surveys Ltd, UK) and hosted on a secure remote server.

**Figure 2:**
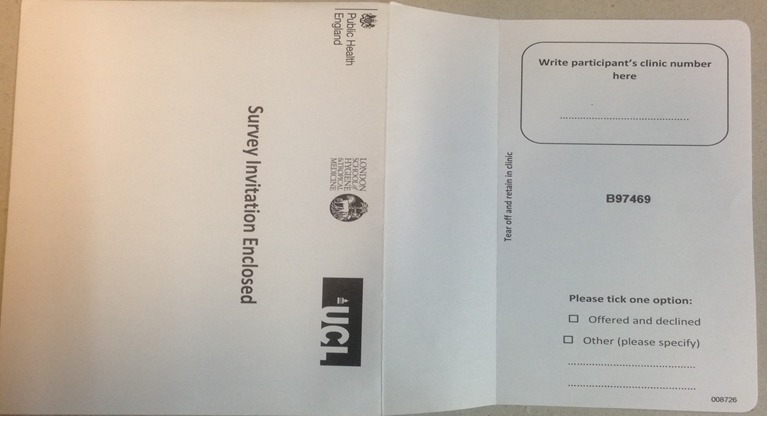
Survey invitation envelope: back side with a tear-off tab.

Therefore, internet connectivity would be required in SHCs to enable attendees to log in to the survey and for the data to upload automatically to a remote server. The study team would provide SHCs with study envelopes and tablets that could be remotely deactivated in the event of theft. SHC staff would offer all attendees a study envelope, which would contain a PIS, a card with a survey Web link, and a unique study passcode (USP), which they would use to access the survey. The PIS contained study details including information that only the research team would have access to the survey data, their right to withdraw at any point during or before submitting the survey online, the linkage of their survey responses to the data SHCs routinely collect on STI tests, and results collated by PHE for surveillance purposes. Attendees who could not use digital devices or read English would be ineligible for participation. Each envelope would have a detachable receipt with the same USP (Figure 2). For those who would agree to participate, clinic staff would retain the detachable receipt and write the participant’s clinic number on it to enable linkage of their survey data to their EHR. For attendees who declined to participate, staff would document that on the detachable receipt and retain the entire envelope. Staff would then enter these data into recruitment sheets to be shared monthly with the researchers.

**Figure 3:**
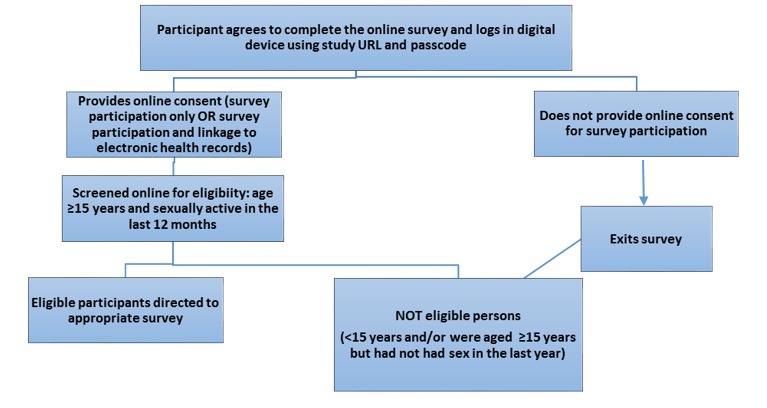
Screening process to identify participants eligible to complete the Web-based survey. EHR: electronic health records.

On logging into the survey, participants would again be shown online the same PIS given to them in the study envelope. They would then be asked to give online consent separately for survey participation and linkage of their survey data to the EHR. If they declined to participate, they would exit the survey (Figure 3). If they declined to linkage, they could participate in the survey, but their responses would not be linked to EHR. Subsequently, they would be screened for eligibility and exit the survey if they reported that they were aged <15 years or ≥15 years but had not had sex in the last year. During online screening, in combined clinics all participants aged ≥16 years who reported having male or male as well as female sexual partner(s) in the last 12 months were directed to the MSM survey and the others were directed to the heterosexual survey. In MSM only clinics, participants who were <16 years, reported having sex only with female partner(s) in last 12 months were excluded.

Subsequently, eligible participants would complete the Web-based survey, which was designed to take approximately 10 to 15 min depending on the sections of the survey that were applicable to them. On completion, researchers could download the participants’ survey data from the server and link these to the EHR (for consenting participants only), using the participant’s clinic number and USP recorded by the staff, to create a study dataset. We anticipate that the BBEST would be implemented periodically in SHCs or settings that routinely collect GUMCAD data to provide in-depth intelligence on issues of particular public health concern, including STI outbreaks.

### Qualitative Study

From June 2014 to August 2015, face-to-face or telephone interviews were conducted with 20 staff members recruited from all study sites to assess the acceptability of the proposed BBEST model. A total of 61 MSM were recruited to 1 of the 8 focus group discussions (FGDs) with the help of lesbian, gay, bisexual and trans/sexual health community-based organizations (CBOs) via newsletters and Facebook pages and an MSM geospatial sociosexual networking application [12]. Moreover, 65 BC participants (n=32 men), aged 15-70 years, recruited from SHCs, colleges, and CBOs, participated in 5 FGDs and 31 interviews. All participants were given a PIS containing study details, including information about who would have access to the data and the participants’ right to withdraw at any point during or at the end of the interviews and FGDs, and then written informed consent was obtained. Piloted topic guides were used during the FGDs and interviews. Participants were shown printouts of the study envelope (Figure 2), card with a survey Web link and USP, and diagrammatic presentation of the proposed linkage procedure to assess feasibility and acceptability of these processes. All the interviews and FGDs were audio-recorded and transcribed verbatim.

### Phase 2: Assessing the Feasibility of Implementing the Bio-Behavioral Enhanced Surveillance Tool

#### Web-Based Surveys

Two separate Web-based questionnaires were developed, because of differences in STI epidemiology in MSM and BC populations [14], and cognitively tested [15]. One of the surveys was designed to be administered in BC only study sites, and the other in MSM only and combined study sites. Between February and April 2016, the proposed model of BBEST was piloted in 4 SHCs. Subsequently, between May and September 2016, all study sites invited clinic attendees to complete the survey, either in the clinic or at home.

### Interviews With Clinic Staff

After completing survey recruitment, short, audio-recorded, semistructured face-to-face/telephone interviews were conducted, between December 2016 and February 2017, with 25 SHC staff from study sites to understand their experiences of implementing the BBEST.

### Linkage of Survey Data to Electronic Health Records

Deterministic and probabilistic methods were used to match records in the survey data and GUMCAD using the following key variables: participants’ clinic number, age, gender, and clinic attendance date. Probabilistic methods allowed matching of records with erroneous or missing data based on minor discrepancies in age, attendance date, and clinic number. After matching, participants’ clinic numbers were dropped to create an anonymous dataset.

### Analysis

Phase 1 and phase 2 qualitative data were thematically analyzed using NVivo 11 for Windows (QSR International Pty Ltd, Australia) to assess acceptability of the proposed BBEST model and to examine barriers and facilitators to implementing the BBEST in SHCs, respectively. We used the Framework method to thematically analyze qualitative data [16]. Accordingly, first, we coded the data according to the key areas explored in the topic guides. Subsequently, we coded data for each key topic of interest, for example, all data coded as “digital device for survey,” was retrieved, and analyzed to identify themes that summarized participants’ common and divergent views concerning the acceptability and feasibility of using digital devices for survey completion, and an index of themes was developed and applied to the qualitative dataset.

The feasibility of implementing the BBEST in phase 2 was assessed quantitatively by examining the recruitment sheets for the number of attendees who were invited to participate in the Web-based survey. The study dataset was examined for the total number of attendees who actually logged in and gave online consent for survey participation and for linkage and the number of eligible participants who completed the survey. The Snap survey software metadata were examined to determine whether a study tablet or other device was used for survey completion. The feasibility of linkage was determined from the number of surveys that were successfully linked to EHR for those who had consented to linkage. Univariable logistic regression was used to examine the association between consent to linkage and the sociodemographic characteristics of survey participants. Representativeness of the survey sample was ascertained by comparing the sociodemographic and sexual health characteristics (only for participants who had consented to and could be linked to their EHR) of the survey participants recruited from the BC only and combined study sites with all the SHC attendees during the study period (data extracted from GUMCAD) using z-test for proportions. The sample recruited from the MSM-only study sites was excluded from this analysis because it was not expected to be representative of “all” SHC attendees due to the study eligibility criteria in these sites (ie, the exclusion of all women and men reporting only female sex partner(s) in the last 12 months; Figure 3). All men identifying as gay/bisexual recruited from all the study sites were compared with all gay/bisexual men accessing these SHCs during the study period to ascertain the representativeness of MSM sample. Stata v13 was used for quantitative data management and analysis.

## Results

### Acceptability of the Proposed Bio-Behavioral Enhanced Surveillance Tool Model

#### Perceived Barriers and Facilitators to Implementing Bio-Behavioral Enhanced Surveillance Tool Among Clinic Staff

##### Internet/Wi-Fi Connectivity

The lack of internet/Wi-Fi connectivity required to administer the Web-based survey and upload the data automatically to a remote server was one of the most commonly perceived barriers by the clinic staff to implementing the BBEST. Staff from clinics located in areas of high deprivation felt that participants may be reluctant to use their own smartphones for survey completion if free Wi-Fi was unavailable in the clinics.

##### Logistics of Using Tablets for Survey

Clinic staff expressed inability to monitor the security of the tablets because of work pressures and challenges in having dedicated staff members to offer tablets to participants. Although some clinics had separate research staff to help with recruitment, other clinics felt that they would have to depend on regular clinic staff, which was perceived as challenging due to staff cuts that were taking place during our study in several clinics and their high workload. Several research studies being undertaken in the clinic simultaneously were also perceived as a potential barrier. A need to seek help from local clinical research networks (CRNs) who provided temporary staff support for research was identified.

##### Lack of Experience of Using Digital Devices

Some clinic staff expressed anxiety about using tablets for administering the survey because of their lack of/limited experience of using either tablets or the internet or both. They expressed a need for training to use the tablets and administer the survey.

##### Documenting Participant’s Clinic Number With a Unique Passcode for Linkage to Electronic Health Records

One of the clinics had concerns about sharing the clinic numbers of survey participants who had not agreed for linkage of their survey responses to EHR, but were willing to provide clinic numbers of participants who had given consent to linkage.

#### Acceptability of Bio-Behavioral Enhanced Surveillance Tool Among Men Who Have Sex With Men and Black Caribbean Participants

Overall, there were few differences in the perceived acceptability of the BBEST among MSM and BC participants, with similar views being expressed by interview and FGD participants.

##### Using Digital Devices for Survey Completion: Confidentiality

Majority of the participants expressed an ability and willingness to use a digital device to self-complete a Web-based survey because it was considered to be potentially confidential due to immediate online submission of responses post survey completion. Web-based surveys were also perceived to be less embarrassing than a face-to-face paper questionnaire because of the lack of potential for clinic staff to read participants’ survey responses. Using a personal digital device compared with a device offered by the clinic for survey completion was preferred because of concerns about applications that may be installed on clinic devices and its impact on confidentiality:

Int: And would you be willing to complete this survey on your own device, if you had one, which had access to the internet?

IDI_001: I’d feel more comfortable doing it on my own device than something that was given to me.

Int: And why do you say that?

IDI_001: Because I don’t know what else that device has on it, whereas I know what my device has on it. So, like, there are apps that can log key strokes, for example, and stuff like that, so again I’d be trusting that device and that person that gave me that device.

Int: Sure.

IDI_001: Whereas if it was just a URL and a pass code and I could use my own device, I’d feel much more comfortable doing that.

BC female interview participant, aged 35 years, Birmingham

Unlike the BC participants, majority of the MSM were familiar with completing Web-based surveys on their phones and considered it to be a secure and efficient method and had greater preference for single or multiple-choice tick-box questions. However, they were unwilling to download a survey app on their phone. Nevertheless, some participants were not willing to use their personal device for survey completion if they did not have access to free Wi-Fi in the clinic:

Respondent 6 Group 5: I’ve found, because I’ve done quite a few of these. When you do one, for some reason slightly straight away, once you’ve done one, this is easy they give you more to do. It almost becomes like great fun.

Int: Okay, that’s what it feels like?

Respondent 6 Group 5: I, I personally find, I’m just talking about me now as an individual, is the multiple choice questions (you) touch on an iPhone, iPad, I love them. I love them because I don’t mind.

Int: Okay, sure.

Respondent 6 Group 5: But I really hate when you have to type in open-ended (answers).

MSM FGD participant, aged 37 years, Manchester

Respondent 5 Group 5: I think you run the risk of people not doing it. You know if it’s there in paper form or iPad form or whatever, I’d do it. But I know full well I would leave, probably do the shopping, get the iPad out, life would kick in.

MSM FGD participant, aged 22 years, Manchester

##### Using Digital Devices for Survey Completion: Prior Experience

Participants with experience of completing Web-based surveys perceived it to be a time-saving method because of routing to subsequent questions being informed by their responses to previous questions. However, a few participants expressed an inability to complete a Web-based survey because they did not know how to use the internet, although some were willing to do so if they were shown how to use the digital devices for survey completion:

Respondent 1 Group 3: And obviously you can ask questions that, you know…or miss questions. You don’t need to ask seventeen questions.

MSM FGD group participant, aged 36 years, Leeds

Respondent 2 Group 3: You only get the ones pertinent to you.

MSM FGD group participant, aged 46 years, Leeds

##### Venue for Survey Completion: Ease and Privacy

Many participants expressed a preference to complete the survey in the clinic because of concerns of getting busy with other things once home. The need to log on to a personal computer once home was perceived to be time-consuming, and not living alone was perceived as a barrier to privacy. However, some participants expressed a preference to complete the survey at home to allow for more considered responses and privacy. Private clinic rooms/booths were preferred for survey completion compared with crowded waiting rooms. Some participants suggested tailoring survey recruitment to the patients flow through the clinic or the layout of services to reduce anxiety about losing their place in the clinic appointment queue:

Int: And do you think, if you were to take part in the survey, you’d rather it all happened in the clinic, rather than doing it in your own time at home?

IDI-17: Well, not necessarily, it’s just when you get home, you’ve got to make something to eat, you’ve got to do all this kind of stuff, and then you need to get your computer out and log in and all this kind of stuff, so it’s that kind of impetus to do that really.

Int: Okay.

IDI-17: Whereas if you get given a tablet which is secure obviously, and it’s there set up for you ready, then it’s a lot kind of easier.

BC male interview participant, aged 44 years, London

##### Linking Survey Responses to Electronic Health Record: Anonymity

Overall, most participants were supportive of the proposed linkage of survey data to EHR because they considered it to “be for something constructive” like improving health care. But some participants perceived it as “too much information gathering on people.” Thereby, providing anonymous online consent, separately for linkage to EHR, was perceived to be acceptable. The proposed usage of a USP as opposed to identifiable details to access the survey was considered important to ensure anonymity.

### Feasibility of Implementing the Bio-Behavioral Enhanced Surveillance Tool

The above-mentioned findings informed the development of site-specific BBEST models. Temporary staff from the local CRN, who are funded by UK Health Department to provide infrastructural support for patient benefit–related research [17], were arranged to help with the implementation of the study procedures in clinics without dedicated research staff (n=10). In other SHCs, existing and temporary CRN staff (n=2) or preexisting research or administrative staff or both (n=4) were arranged to do so. All staff involved with administering the study were trained by the researchers to implement the standard operating protocol and to use the tablets. Each participating SHC was provided with at least one 3G-enabled iPad and a cable and lock to ensure its security.

#### Recruitment Offer and Survey Completion Rates

**Figure 4:**
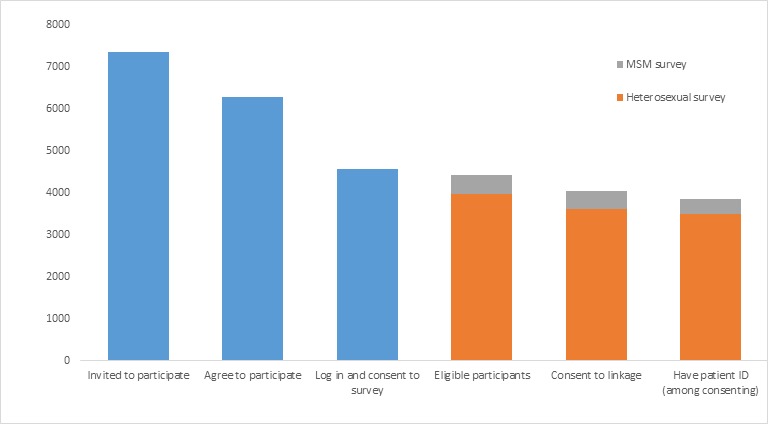
Feasibility of implementing the Bio-Behavioral Enhanced Surveillance Tool by population of interest. MSM: men who have sex with men.

**Figure 5:**
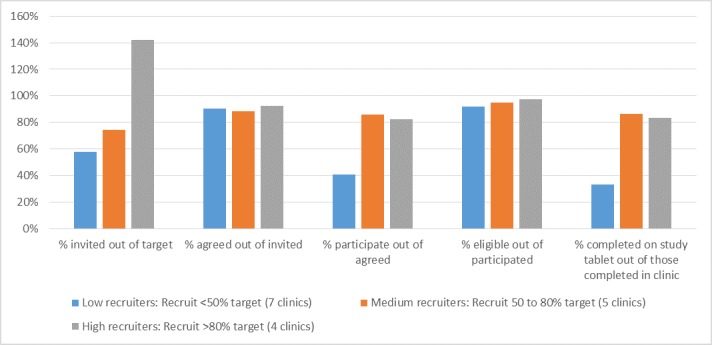
Recruitment cascade among clinics grouped by recruitment success.

Of the 7367 attendees recorded by SHCs as invited, 6283 (85.28%) agreed to participate (Figure 4); 73.62% (4626/6283) logged in, of whom 21.59% (999/4626) did so using their personal device, and 72.97% (4585/6283) consented to participate in the survey. Moreover, 70.63% (4437/6283) of those who agreed to participate were eligible. However, recruitment success between SHCs varied considerably. Of clinic-specific recruitment targets, 7 SHCs recruited <50% (categorized as “low recruiters”), 5 SHCs recruited between 50% and 80% (“medium recruiters”’), and 4 SHCs recruited >80% (“high recruiters”). Considerably, more clinic attendees were invited to participate in the survey by high recruiters (median 142.23% of the clinic-specific target) compared with low and medium recruiters (57.91% and 74.13%, respectively; Figure 5). Although the proportion of those invited who agreed to participate did not vary between these 3 types of SHCs and ranged between 91% and 93%, the proportion of participants who actually logged in and consented to participate in the survey was higher in medium (85.30%) and high (81.79%) recruiting SHCs than low (40.70%) recruiting SHCs. Similarly, a higher proportion of participants who completed the survey in the clinic in medium (86.19%) and high (83.60%) recruiting SHCs than low (33.12%) recruiting SHCs used a study tablet to log into the survey. There was no difference in the proportion of participants who were eligible for the survey across these groups (range: 92%-97%).

#### Barriers and Facilitators to Implementing the Bio-Behavioral Enhanced Surveillance Tool

Phase 2 staff interviews highlighted that SHC with a culture of valuing and prioritizing research and innovation, championed by senior clinicians, was an important factor for successfully implementing the BBEST. Having enthusiastic staff members, especially with dedicated roles and time to implement the study, maximized the recruitment. However, structural changes in clinical services, including a London-wide reorganization of sexual health, negatively affected staff morale and their engagement with the study, and in some SHCs, it led to frequent staff turnover. This affected recruitment with subsequent staff receiving limited training in study procedures, resulting, for example, in one clinic not documenting participants’ clinic numbers. Nevertheless, despite initial concerns, the majority of staff involved with implementing the survey became familiar and gained confidence with the use of tablets. However, some clinics offered tablets to participants only if they had dedicated staff to monitor them because of concerns of theft or damage. One tablet was stolen from a locked cabinet in a staff-only access area, and in 2 clinics, the lack of ability to secure tablets to immovable objects prevented their use. The limited number of tablets available per clinic for administering the survey restricted their ability to recruit multiple participants simultaneously. However, in clinics that had Wi-Fi, the option for participants to complete the survey using their own digital device was considered a facilitator for recruitment. Some staff felt overburdened with the requirement to record participants’ clinic number and sending the recruitment sheet monthly to the research team. Nonetheless, they appreciated the real-time feedback from the study team on survey completion rates, which enabled them to promote completion in the clinic, as opposed to home, to address lower home completion rates.

#### Feasibility of Linking Survey Data to Electronic Health Records

Altogether, 91.19% (4046/4437) of the eligible participants consented to link their survey data to EHR (Figure 4). This did not differ by age or gender but was higher among gay/bisexual than heterosexual men (95.50%, 722/756 vs 88.31%, 1073/1215; *P*<.003) and lower among BC than white participants (87.25%, 568/651 vs 93.89%, 2181/2323; *P*<.002). Of those participants who had consented to linkage, 88.88% (3596/4046) of surveys were successfully linked to EHR (80.98%, 3593/4437, of eligible participants). Moreover, 83.49% (3000/3593) of these records matched on all the variables, with the remaining matches differing slightly in at least 1 variable, including 3.23% with typographical errors in the USP. In addition, 34.7% (156/450) surveys could not be linked to EHR because the staff did not record either the USP or the participants’ clinic number. The remaining surveys could not be linked either because of errors in the clinic number that could not be resolved using the probabilistic linkage algorithm or a mismatch between the participant’s clinic number used in the clinic compared with that submitted to GUMCAD.

**Table 1:** Comparison of sociodemographic characteristics of survey participants with all clinic attendees during the study period.

Sociodemographic characteristics and indicators of sexual health			GUMCAD data on attendees in clinics administering only heterosexual survey (N=97,054)		Survey population in clinics administering only heterosexual survey (N=4184)		*P* value (Z test for proportions comparing nonmissing)
			n (%)	Percentage excluding unknown/missing variables	n (%)	Percentage excluding unknown/missing variables	
**Gender**							
	Male		41,609 (42.87)	42.87	1722 (41.16)	41.16	.03
	Female		55,411 (57.09)	57.09	2447 (58.48)	58.48	.08
	Trans/other^a^		Not available	Not available	15 (0.36)	0.36	Not calculated
	Unknown/missing		34 (0.00)	—^b^	0 (0.00)	0.00	—
**Sexual orientation (of all men)**			**N=41,609**		**N=1722**		
	Gay/bisexual		9597 (23.06)	23.90	519 (30.14)	31.52	<.001
	Heterosexual		30,585 (73.51)	76.10	1165 (67.65)	70.61	<.001
	Unknown		1427 (3.43)	—	73 (4.24)	—	—
**Age, in years**							
	<25		30,034 (30.94)	30.94	1,599 (38.21)	38.21	<.001
	≥25		67,020 (69.05)	69.05	2585 (61.78)	61.78	<.001
	Unknown/missing		0 (0.00)	—	0 (0.00)	—	—
**Ethnicity**							
	White		49,409 (50.91)	56.00	2092 (50.00)	51.20	<.001
	Black African		7960 (8.19)	9.01	418 (9.99)	10.2	<.001
	Black Caribbean		8368 (8.62)	9.51	640 (15.29)	15.7	<.001
	Black other		4441 (4.60)	5.01	42 (1.00)	1.00	<.001
	Mixed		6549 (6.78)	7.41	370 (8.80)	9.11	<.001
	Asian		8224 (9.29)	9.29	400 (9.62)	9.81	.32
		Other ethnicities	3288 (3.38)	3.38	125 (2.98)	2.98	.25
	Unknown/missing		8815 (9.08)	—	97 (2.31)	—	—
**Sexual health outcomes using total linked data for clinics that offered heterosexual survey^c^**					**N=3447**		
	**Sexual health screen on day of clinic attendance^d^**						
		Yes	74,217 (76.52)	76.52	2552 (73.98)	73.98	<.001
		No	22,837 (23.53)	23.53	895 (25.96)	25.96	<.001
	**Diagnosed with an acute STI^e^ on the day of clinic attendance^c,f^**						
		Yes	14,240 (14.67)	14.67	585 (16.97)	16.97	<.001
		No	82,814 (85.32)	85.32	2862 (83.03)	83.03	<.001

^a^Trans not currently recorded on GUMCAD surveillance.

^b^Indicates "not applicable".

^c^There is no “missing” data for these categories as there is no requirement to code when there is no STI screen or STI diagnosis.

^d^Sexual health screen—one of the following test combinations: chlamydia and gonorrhea; chlamydia, gonorrhea, and syphilis; syphilis and HIV; and chlamydia, gonorrhea, syphilis, and HIV.

^e^STI: sexually transmitted infections.

^f^Acute STI—any of chlamydia, gonorrhea, anogenital herpes (first episode), anogenital herpes (first episode), HIV, infectious syphilis, pelvic inflammatory disease/epididymitis, non-specific genital infections, chancroid, lymphogranuloma venereum, donovanosis, trichomoniasis, scabies, pediculosis pubis, molluscum contagiosum, mycoplasma genitalium, shigella, hepatitis A, hepatitis B, and hepatitis C.

**Table 2:** Comparison of sociodemographic characteristics of men who have sex with men (MSM) who participated in the survey compared with all MSM attending all the study sites during the study period.

Sociodemographic characteristics and indicators of sexual health			GUMCAD data (N=11,180)		Survey population (N=751)		*P* value (Z test for proportions comparing nonmissing)
			n (%)	Percentage excluding unknown/missing variables	n (%)	Percentage excluding unknown/missing variables	
**Age, in years**							
	<25		1776 (15.88)	15.88	167 (22.2)	22.2	<.001
	≥25		9404 (84.11)	84.11	584 (77.8)	77.8	<.001
	Unknown/missing		0 (0.00)	0.00	0 (0.0)	0.0	—^a^
**Ethnicity**							
	White		7671 (68.61)	75.00	551 (73.3)	74.3	.70
	Black African		226 (2.02)	2.02	15 (2.0)	2.0	.74
	Black Caribbean		350 (3.13)	3.40	37 (4.9)	5.0	.03
	Black other		132 (1.80)	1.31	2 (0.3)	0.3	.02
	Mixed		487 (4.35)	4.80	50 (6.7)	6.7	.02
	Asian		876 (7.83)	8.61	62 (8.3)	8.3	.85
	Other ethnicities		486 (4.34)	4.81	24 (3.2)	3.2	.06
	Unknown/missing		952 (8.52)	–	10 (1.33)	–	–
**Sexual health outcomes using total linked data for clinics that offered heterosexual survey^b^**					**N=571**		
	**Sexual health screen on day of clinic attendance^c^**						
		Yes	9051 (80.89)	80.89	406 (71.1)	71.1	<.001
		No	2129 (19.04)	19.04	165 (28.9)	28.9	<.001
	**Diagnosed with an acute STI^d^ on the day of clinic attendance^b, e^**						
		Yes	2203 (19.70)	19.70	116 (20.3)	20.3	.72
		No	8977 (80.29)	80.29	455 (79.7)	79.7	.72

^a^Indicates "not applicable".

^b^There is no “missing” for these categories as there is no requirement to code when there is no STI screen or STI diagnosis.

^c^Sexual health screen—one of the following test combinations: chlamydia and gonorrhea; chlamydia, gonorrhea, and syphilis; syphilis and HIV; and chlamydia, gonorrhea, syphilis, and HIV.

^d^STI: sexually transmitted infections.

^e^Acute STI—any of chlamydia, gonorrhea, anogenital herpes (first episode), anogenital herpes (first episode), HIV, infectious syphilis, pelvic inflammatory disease/epididymitis, nonspecific genital infections, chancroid, lymphogranuloma venereum, donovanosis, trichomoniasis, scabies, pediculosis pubis, molluscum contagiosum, mycoplasma genitalium, shigella, hepatitis A, hepatitis B, and hepatitis C.

#### Representativeness of the Survey Sample

Compared with all the SHC attendees accessing BC only and combined study sites during the study period (N=97,054), of the 4184 survey participants recruited from these sites a higher proportion were aged <25 years (38.21%, 1599/4184 vs 30.94%, 30034/97,054; *P*<.001) and were BC (15.29%, 640/4184 vs 8.62%, 8368/97,054; *P<*.001). There was no overall difference in recruitment by sex, but men recruited from these study sites were more likely to identify as gay/bisexual (42.92%, 519/1209 vs 23.06%, 9597/41,609; *P*<.001; Table 1). Responses of 82.39% (3447/4184) of participants’ who were attending clinics that offered the heterosexual survey were linked to their EHR. Compared with all the SHC attendees, a slightly lower proportion of this survey sample had had a same-day sexual health screen during that clinic visit (74.97%, 2552/3447 vs 76.52%, 74,217/97,054; *P<*.001), but a slightly higher proportion of them were diagnosed with acute STIs during that clinic visit (16.97%, 585/3447 vs 14.67%, 14,240/97,054; *P<*.001).

As shown in Table 2, compared with all gay/bisexual identifying men attending SHCs during the study period (N=11,180), a higher proportion of men in the survey sample were <25 years (22.2%, 167/751 vs 15.88%, 1776/11,180; *P*<.001) and were BC (4.9%, 37/751 vs 3.13%, 350/11,180; *P=*.025), but a lower proportion had had a same-day sexual health screen (71.1%, 406/571 vs 80.89%, 9051/11,189; *P<*.001). There was no difference in the proportion who were diagnosed with an acute STI during that clinic visit (20.3%, 116/571 vs 19.70%, 2203/11,180 [*P*=.721]).

## Discussion

### Key Findings

Our findings show that the BBEST is largely acceptable to SHC attendees and staff, and it is feasible to implement in SHCs across England. Specifically, the SHC attendees at greatest STI risk participated in the self-administered Web-based surveys using digital devices and consented to linkage. Linking survey data to the EHR was also feasible. However, a lack of resources dedicated to delivering the BBEST was a barrier to its implementation in some SHCs.

### Strengths and Limitations

Study sites were purposively selected and thus are not representative of SHCs in England. Moreover, the survey offer rates varied considerably across sites increasing the likelihood of recruitment bias. We are unable to fully assess the representativeness of our sample because of the lack of data on decliners, attendees who agreed to participate but did not log in, and those who logged in but did not consent to participate. The representativeness analysis shows that, overall, groups at greater STI risk are overrepresented in the survey, for example, participants aged <25 years, gay/bisexual men, and BC participants. Nevertheless, the proportion of gay/bisexual identifying men who had an acute STI diagnosis on the day of clinic attendance was similar among those in the survey and in the clinic population, highlighting the similar STI risk profile of these men.

### Comparison With Other Studies

Overall, the response rate among those invited to participate was 62.23% (4585/7367); however, survey offer rates between SHCs varied enormously. Similar to our study, this interclinic variation was observed in previous clinic surveys conducted using pen and paper, with response rates among attendees across clinics varying from 41.0% to 70.1% in one study [18] and from 24.9% to 76.1% in another [19]. In both these studies, and as we observed, this variation was attributed to differences in staff’s commitment to, and enthusiasm for, the study and resources available within SHCs for research [18,20]. In our study, CRNs were unable to provide support for recruitment to some SHCs because of a high demand on their resources from multiple studies; however, the majority of sites with CRN support met their recruitment target. Although increasing the CRN support available to clinics may improve their ability to participate in research and thus their response rate, sometimes this may not compensate for “research fatigue,” among clinic staff and clinic attendees from participating in multiple studies taking place in the clinic, which negatively impacts the response.

Compared with another study, a slightly higher proportion of participants in our study consented to linkage of their survey data to their EHR (91.2% vs 84.0%) [20]. This could potentially be due to the cocreation of the BBEST with the involvement of the CAG and SHC attendees and staff. The feasibility of linking the survey data to EHR in our study was high. Similar to findings of a previous study, linkage was unsuccessful in a handful of cases due to minor errors [18]; nevertheless, using a probabilistic method increased match sensitivity.

### Implications for Practice

Our findings suggest that the BBEST can be implemented to collect detailed behavioral data on factors influencing STI risk among SHC attendees, especially among populations at greatest STI risk in the event of outbreaks or periodically in response to significant public health concerns. Furthermore, the BBEST could be implemented in other settings such as hospitals, general practice, and with other populations at STI/HIV risk to strengthen interpretation of existing surveillance data. However, the impact of increasing private health care providers [21,22] on the availability of resources for implementing the BBEST is as yet unknown. Moreover, CRNs provide support only for research studies and not for surveillance activities. Therefore, extension of CRN support to surveillance activities could enhance implementation of BBEST. Although Web-based self-administered surveys were acceptable to SHC attendees, several SHCs did not have a reliable internet connection/Wi-Fi, highlighting the need to rapidly scale up the implementation of plans for digitizing the NHS for patient benefit [23]. Survey software that facilitates real-time data uploading from digital devices to a secure remote server and digital devices that can be remotely deactivated should be used to enhance data security and participants’ confidentiality. PHE does not need SHC attendees’ consent to collect clinical and behavioral data for public health monitoring and response work under section 251 of the UK NHS Act of 2006, although there are strict regulations for doing so [24]. However, obtaining informed consent is considered a norm in health surveys [25] and is particularly important in the context of linking surveillance data to survey data. Our study shows that informed consent can be obtained online, anonymously, and explicitly for survey participation and for linking it to the EHR. In conclusion, in an era of reduced sexual health budgets [22,26] and the limitations of existing routine STI surveillance methods, implementing a BBEST could enable timely collection of detailed behavioral data to better inform effective commissioning of health promotion and STI prevention strategies. However, feasibility of implementing BBEST could be influenced by the availability of resources.

## Abbreviations

BBEST: bio-behavioral enhanced surveillance tool
BC: black Caribbean
CAG: Community Advisory Group
CASI: computer-assisted self-interview
CBOs: community-based organizations
CRN: clinical research network
EHR: electronic health records
FGD: focus group discussions
MSM: men who have sex with men
PIS: participant information sheet
SHC: sexual health clinics
STI: sexually transmitted infections
USP: unique study passcode
